# Supplementary material for: A Scaffold-Free 3-D Co-Culture Mimics the Major Features of the Reverse Warburg Effect In Vitro
Source: Cells. 2020 Aug 13;9(8):1900. doi: 10.3390/cells9081900 (PMC7463893; doi:10.3390/cells9081900)
Supplement: Supplementary file 1 [file cells-09-01900-s001.pdf]

Supplementary figure S1

to manuscript "A scaffold-free 3D co-culture mimics major features of the reverse Warburg effect in vitro" by Florian Keller, Roman Bruch, Richard Schneider, Julia Meier-Hubberten, Mathias Hafner, Rüdiger Rudolf

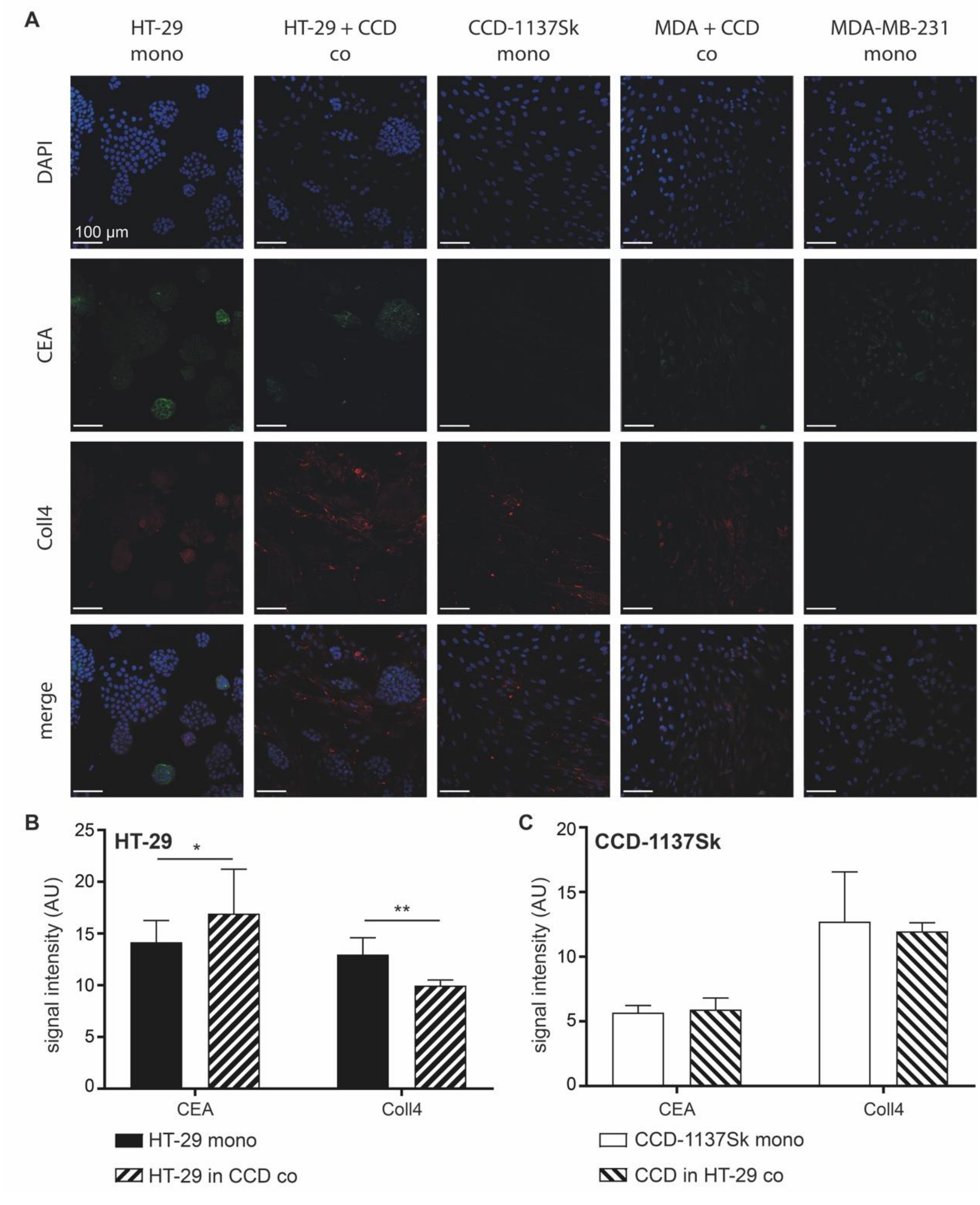

Figure S1: ***CEA can serve as a cancer-cell marker in 2D mono- and co-cultures of HT-29, MDA-MB-231, and CCD-1137Sk cells.*** HT-29, MDA-MB-231, and CCD-1137Sk cells were either seeded alone or in co-culture as indicated and grown to a sub-confluent state for 4 days. Then, cells were fixed and stained with DAPI (blue) as well as antibodies against CEA (green) and Coll4 (red). A) Representative confocal images of fluorescence staining for markers and cultures as indicated. B – C) Quantitative analysis of CEA and Coll4 expression of HT-29 (B) and CCD-1137Sk cells (C). Mean + SEM (n = 3 experiments; * $p < 0.05$, ** $p < 0.01$).

Supplementary figure S2

to manuscript "A scaffold-free 3D co-culture mimics major features of the reverse Warburg effect in vitro" by

Florian Keller, Roman Bruch, Richard Schneider, Julia Meier-Hubberten, Mathias Hafner, Rüdiger Rudolf

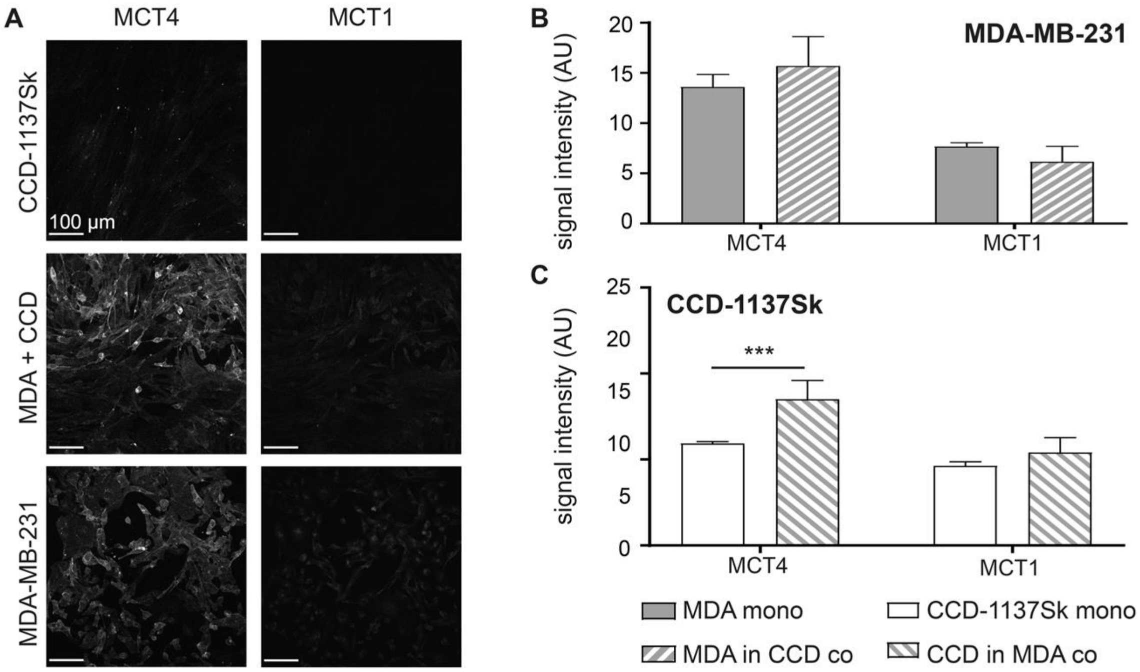

Figure S2: ***Monolayer co-cultures of MDA-MB-231 and CCD-1137Sk show enhanced expression of MCT4 in fibroblasts.*** MDA-MB-231 and CCD-1137Sk cells were either seeded alone or in co-culture and grown to a sub-confluent state for 4 days. Then, cells were fixed and stained with DAPI as well as antibodies against MCT4 and MCT1 as markers for nuclei, lactate export, and lactate import, respectively (A – C). A) Representative confocal images of fluorescence staining for markers and cultures as indicated. B and C) Graphs showing quantitative analysis of fluorescence intensity values for markers and cell type as indicated. Mean + SEM (n = 3 experiments; *** $p < 0.001$).

Supplementary figure S3

to manuscript "A scaffold-free 3D co-culture mimics major features of the reverse Warburg effect in vitro" by

Florian Keller, Roman Bruch, Richard Schneider, Julia Meier-Hubberten, Mathias Hafner, Rüdiger Rudolf

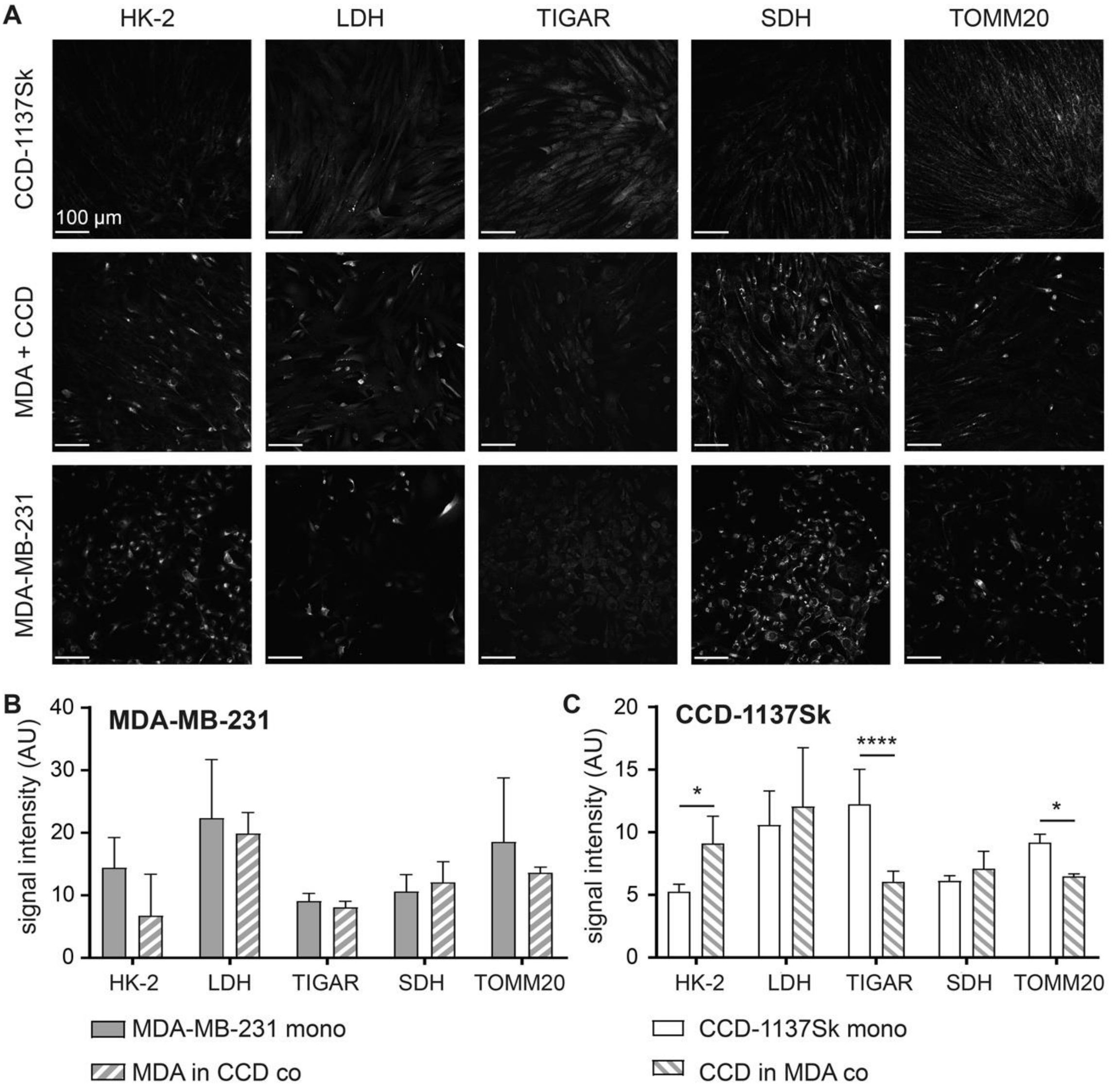

Figure S3: ***Fibroblasts in monolayer co-cultures of MDA-MB-231 and CCD-1137Sk show enhanced glycolytic and reduced oxidative phosphorylation markers.*** MDA-MB-231 and CCD-1137Sk cells were either seeded alone or in co-culture and grown to a sub-confluent state for 4 days. Then, cells were fixed and stained with DAPI as well as antibodies against HK-2, LDH, TIGAR, SDH, and TOMM20 as markers for nuclei, glucose breakdown, pyruvate-lactate metabolism, negative glycolysis regulation, oxidative phosphorylation, and mitochondrial content, respectively (A – C). A) Representative confocal images of fluorescence staining for markers and cultures as indicated. B and C) Graphs showing quantitative analysis of fluorescence intensity values for markers and cell type as indicated. Mean + SEM (n = 3 experiments; * $p < 0.05$, **** $p < 0.0001$).

Supplementary figure S4

to manuscript "A scaffold-free 3D co-culture mimics major features of the reverse Warburg effect in vitro" by Florian Keller, Roman Bruch, Richard Schneider, Julia Meier-Hubberten, Mathias Hafner, Rüdiger Rudolf

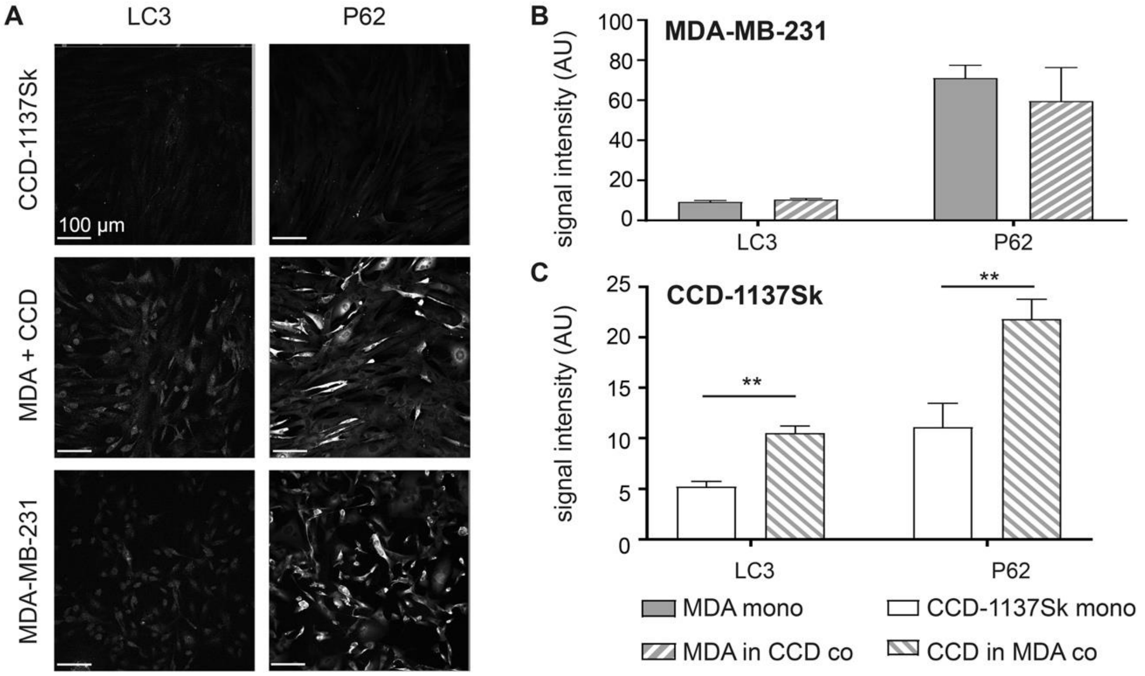

Figure S4: ***Fibroblasts in monolayer co-cultures of MDA-MB-231 and CCD-1137Sk display enhanced levels of autophagy markers.*** MDA-MB-231 and CCD-1137Sk cells were either seeded alone or in co-culture and grown to a sub-confluent state for 4 days. Then, cells were fixed and stained with DAPI as well as antibodies against LC3 and P62 as markers for nuclei and autophagy (A – C). A) Representative confocal images of fluorescence staining for markers and cultures as indicated. B and C) Graphs showing quantitative analysis of fluorescence intensity values for markers and cell type as indicated. Mean + SEM (n = 3 experiments; ** $p < 0.01$).

Supplementary figure S5

to manuscript "A scaffold-free 3D co-culture mimics major features of the reverse Warburg effect in vitro" by Florian Keller, Roman Bruch, Richard Schneider, Julia Meier-Hubberten, Mathias Hafner, Rüdiger Rudolf

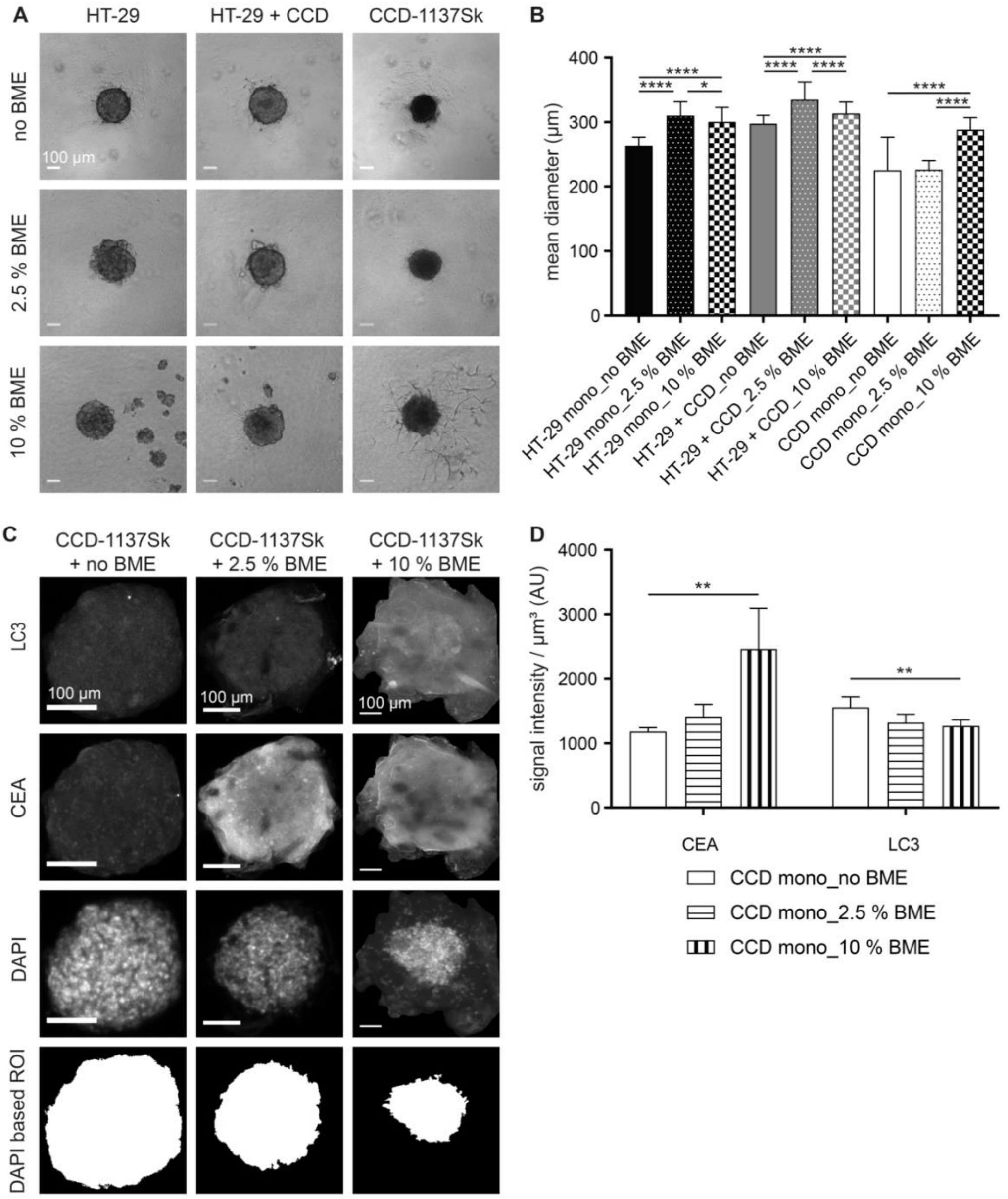

Figure S5: ***Culture-embedding in BME affects growth and fluorescence properties of spheroids.*** HT-29 and CCD-1137Sk cells were either seeded alone or in co-culture in the absence or presence of 2.5 % or 10 % BME to form spheroids. After 4 days, cultures were examined with bright-field microscopy (A) to determine spheroid diameters. Note extensive cellular anastomoses in CCD-1137Sk cultures with 10 % BME. B) Graph showing average spheroid diameters. Mean + SEM (n = 90; * p< 0.5, **** p < 0.0001). C and D) Upon wide-field microscopy, 3D cultures were fixed, stained with DAPI, anti-CEA, and anti-LC3 and then images with confocal microscopy. C) representative sum-z projections of confocal image stacks for conditions and markers as indicated. Bottom panels show regions of interest (ROI) selected for quantitative image analysis. These contain the core spheroids with most of the nuclei. However, particularly the condition with 10 % BME shows a wide halo with scattered cells that were hard to distinguish and were therefore excluded from analysis. D) Quantitative analysis of the sum-projections. Mean + SEM (n = 5 experiments; ** p < 0.01).

Supplementary figure S6

to manuscript "A scaffold-free 3D co-culture mimics major features of the reverse Warburg effect in vitro" by Florian Keller, Roman Bruch, Richard Schneider, Julia Meier-Hubberten, Mathias Hafner, Rüdiger Rudolf

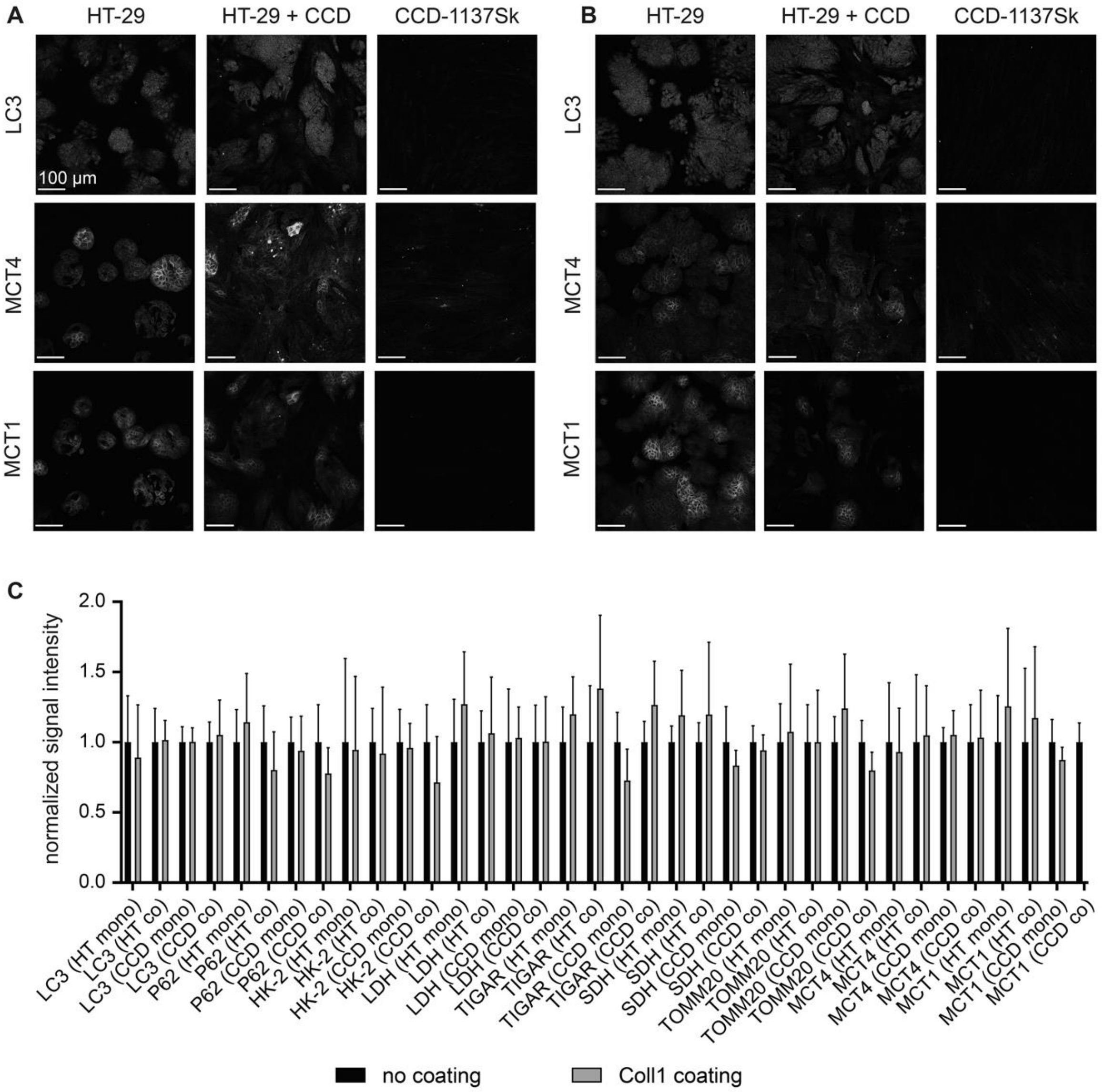

Figure S6: ***Surface coating with Collagen 1 does not alter the expression patterns of several metabolic markers.*** HT-29 and CCD-1137Sk cells were either seeded alone or in co-culture and grown to a sub-confluent state for 4 days. Then, cells were fixed and stained with DAPI as well as antibodies against several markers as indicated. A and B) Representative confocal images of fluorescence staining for markers LC3, MCT1, and MCT4 of cultures as indicated in the absence (A) and presence (B) of Collagen-I coating. C) Graph showing quantitative analysis of fluorescence intensity values for markers and cell type as indicated. All values are normalized to the corresponding average value measured in the absence of coating with Collagen1. Mean + SEM (n = 3 experiments). None of the differences was significant.
